# Autopolyploidization reshapes transcription factor regulatory networks and enhances MAPK-associated thermotolerance in rice

**DOI:** 10.3389/fpls.2026.1813232

**Published:** 2026-04-16

**Authors:** Changjiang Zhang, Yu Wang, Xiaoyu Wang, Honglin Yuan, Yingkai Wang, Weilong Meng, Minghong Xu, Jian Ma, Ningning Wang

**Affiliations:** 1 Faculty of Agronomy, Jilin Agricultural University, Changchun, China; 2 SanJiang Laboratory, Changchun, China

**Keywords:** autopolyploidization, gene co-expression network, heat stress, MAPK signaling, rice (*Oryza sativa* L.), transcription factors

## Abstract

Polyploidization is an important evolutionary mechanism that contributes to plant adaptation to environmental stresses; however, the transcriptional regulatory mechanisms underlying enhanced thermotolerance in autopolyploid crops remain poorly understood. In our previous work, we demonstrated that the superior thermotolerance of tetraploid rice compared with diploid rice under heat stress was associated with DNA methylation and accompanied by differential expression of related genes. Nevertheless, the underlying molecular mechanisms remain to be further elucidated. In this study, we compared diploid japonica rice (GFD-2X) and its naturally derived autotetraploid counterpart (GFD-4X) under control, heat stress, and recovery conditions to investigate transcription factor (TF)-centered regulatory networks. Transcriptomic analysis identified 1,141 expressed TF genes across 56 families. Heat stress induced widespread transcriptional repression in both genotypes, with approximately 70% of differentially expressed TF genes (DETFs) downregulated. However, the autotetraploid genotype exhibited nearly threefold more genotype-specific DETFs under stress and demonstrated pronounced transcriptional reactivation during recovery, with upregulated DETFs exceeding downregulated genes by more than twofold. Family-level analysis revealed a regulatory shift from bHLH-dominated regulation during acute heat stress to WRKY- and MYB-associated modulation during recovery. Functional enrichment analysis highlighted hormone-mediated signaling and the mitogen-activated protein kinase (MAPK) signaling pathway as central regulatory components. Weighted gene co-expression network analysis (WGCNA) identified four modules positively associated with the autotetraploid genotype and 67 hub genes, including five MAPK-associated TFs (*OsbZIP23, OsbZIP49, OsbZIP84, OsRR26*, and *OsMYC2*) forming a coordinated regulatory core. These findings suggest that autopolyploidization enhances thermotolerance by restructuring TF-centered regulatory networks, reinforcing MAPK–hormone signaling integration, and strengthening recovery-phase transcriptional reprogramming. Our study provides network-level insights into how genome duplication promotes stress resilience in rice.

## 1 Introduction

Whole-genome duplication (WGD), or polyploidization, is a pervasive evolutionary event in plants and has played a fundamental role in species diversification, adaptation, and crop domestication (Shimizu-Inatsugi et al., 2017; Fan et al., 2022; Heslop-Harrison et al., 2023; Twyford et al., 2025). Many major crops, including rice and wheat, have experienced genome duplication events during their evolutionary history (Salman-Minkov et al., 2016; Ramírez-González et al., 2018). Polyploidization can generate genetic redundancy, alter gene dosage balance, and promote regulatory divergence, thereby conferring adaptive advantages under fluctuating environmental conditions (Song and Chen, 2015; Wang et al., 2022). In rice, previous studies have demonstrated that autotetraploid lines exhibit improved tolerance to abiotic stresses such as salinity, accompanied by altered expression of hormone- and oxidative stress-related genes (Yong et al., 2023). However, the transcriptional regulatory mechanisms underlying enhanced thermotolerance in autotetraploid rice remain largely unresolved.

Heat stress is one of the most severe abiotic stresses affecting global crop productivity. Elevated temperatures disrupt membrane integrity, impair enzyme activity, and reduce photosynthetic efficiency, ultimately limiting growth and reproductive success (Guilioni et al., 2003; Larkindale and Huang, 2004; Jagadish et al., 2007). In cereals such as wheat and rice, high temperature during reproductive stages can reduce grain filling and fertility, leading to significant yield losses (Matsui et al., 2000; Mas‐ud et al., 2025). To mitigate heat-induced damage, plants have evolved complex defense systems that integrate stress perception, signal transduction, and transcriptional reprogramming (Jiayu, 2025; Li et al., 2025a). These responses often involve rapid activation of heat shock proteins and other protective genes, coordinated by upstream signaling pathways and transcription factors (TFs).

Transcriptional regulation represents one of the central mechanisms by which plants perceive and respond to environmental stresses (Hamazaki et al., 2021). As core components of transcriptional regulatory networks, TFs play pivotal roles in coordinating gene expression. TFs are proteins characterized by conserved structural domains that enable the precise recognition and binding of specific nucleotide sequences located in the 5′ upstream regulatory regions of target genes, such as promoters or enhancers, thereby modulating gene transcription (Li et al., 2025b). When environmental conditions change, TFs function as critical signal transducers, converting external stimuli into intracellular signaling events. Through dynamic regulation in a spatiotemporal and dosage-dependent manner, they activate or repress the transcription of downstream target genes and consequently influence subsequent protein synthesis. This finely tuned regulatory capacity ultimately shapes plant growth and development, as well as adaptive responses to abiotic and biotic stresses (Sukumari Nath et al., 2019; Feng et al., 2020).

Several TF families have been implicated in heat and other abiotic stress responses, including AP2/ERF, bZIP, MYB, WRKY, NAC, and bHLH (Xie et al., 2019; Du et al., 2023). For example, OsMYB55 enhances heat tolerance in rice by regulating proline biosynthesis and reducing reactive oxygen species accumulation (Pei et al., 2017). In maize, ZmNF-YA9 improves heat tolerance by enhancing reactive oxygen species (ROS) scavenging and maintaining photosystem stability (Siting, 2025). Similarly, AP2/ERF family members have been shown to regulate abiotic stress tolerance in multiple crops (Xiusheng, 2025). These findings highlight the central role of TF-mediated regulatory networks in plant heat adaptation.

Despite substantial progress in understanding individual TF functions, the relationship betweengenome duplication and stress-responsive transcriptional network architecture remains poorly characterized. Polyploidization may influence stress tolerance not only through increased gene copy number but also through reorganization of regulatory networks and altered signal integration. Previous physiological studies suggest that polyploid plants often display enhanced osmotic adjustment, antioxidant capacity, and cellular stability under stress (López-Jurado et al., 2021; Ng et al., 2018). In our previous study, we investigated the mechanistic differences between diploid and autotetraploid rice in response to heat stress, revealing how polyploidization, together with dynamic changes in DNA methylation, coordinately regulates thermotolerance in rice. We further elucidated the central roles of hormone signaling, the antioxidant system, and heat shock proteins (HSPs) in this process (Zhang et al., 2026). However, it remains unclear whether these phenotypic advantages are associated with coordinated rewiring of TF networks, particularly across both stress and recovery phases.

In this study, we compared diploid japonica rice (GFD-2X) and its naturally derived autotetraploid counterpart (GFD-4X) under control, heat stress, and recovery conditions. By focusing on TF-centered transcriptomic analysis, differential expression profiling, functional enrichment, and weighted gene co-expression network analysis (WGCNA), we aimed to elucidate how polyploidization reshapes regulatory architecture under heat stress. We hypothesized that autotetraploid rice exhibits enhanced thermotolerance through coordinated remodeling of mitogen-activated protein kinase (MAPK)–hormone signaling pathways and TF networks, leading to expanded regulatory plasticity and improved recovery-phase transcriptional reactivation. Our findings provide new insights into the network-level mechanisms by which genome duplication enhances stress resilience in crops and identifies candidate regulatory genes for future breeding and functional validation.

## 2 Results

### 2.1 Global transcription factor landscape reveals treatment-dominant clustering and ploidy-dependent basal regulatory divergence

To investigate transcriptional responses to heat stress, we performed RNA sequencing (RNA-seq) onleaf tissues of the japonica rice GFD diploid (GFD-2X) and its homologous tetraploid (GFD-4X) under control, heat stress, and recovery conditions. Each treatment included three independent biological replicates. Following the heat stress treatment regime, samples grown under normal conditions for 7 days were designated as Mock_2X and Mock_4X. Samples exposed to high-temperature treatment for 7 days were labeled HT_2X and HT_4X. For the recovery phase, plants grown under normal conditions for 14 days were designated R_Mock_2X and R_Mock_4X, whereas plants subjected to heat stress followed by 7 days of recovery under normal conditions were labeled R_HT_2X and R_HT_4X. For RNA-seq analysis, sequencing quality was high across all samples. The overall sequencing error rate was below 0.03%, the GC content of clean reads exceeded 51.21%, and Q30 values were greater than 92.76% (**Supplementary Table 1**), indicating the reliability and robustness of the transcriptomic dataset.

**Figure 1:**
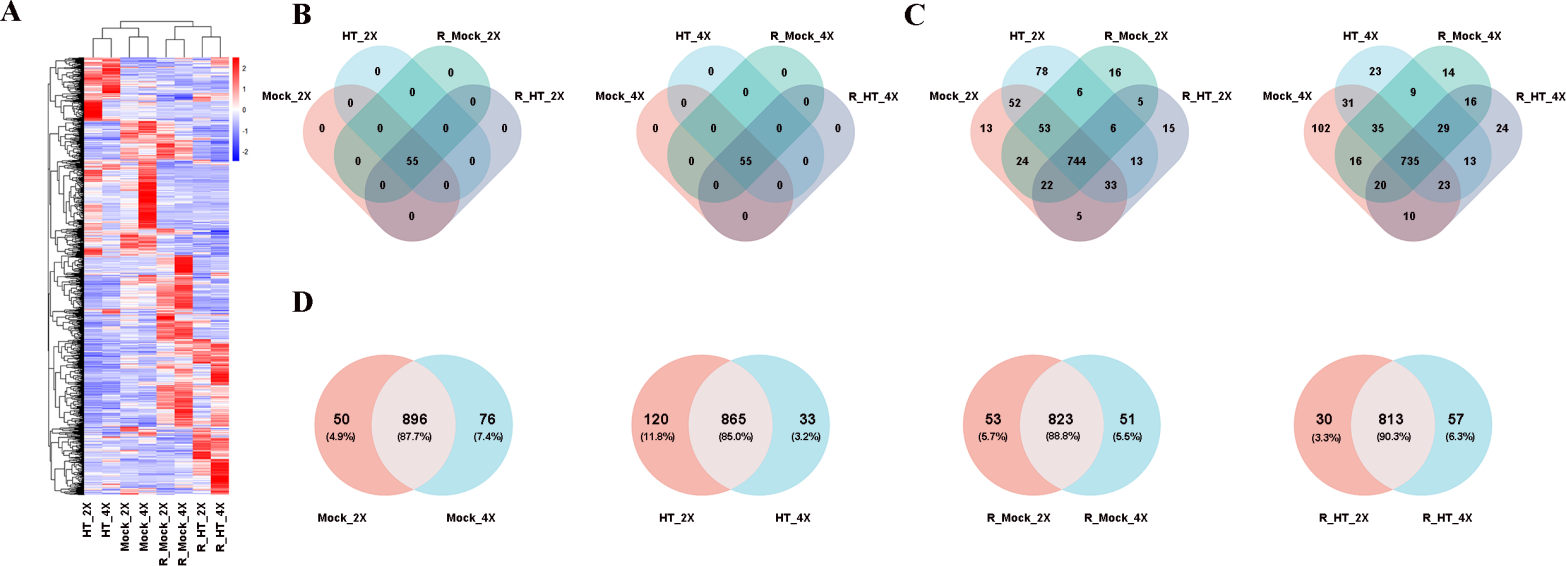
Global TF, transcription factor expression landscape in diploid (GFD-2X) and autotetraploid (GFD-4X) rice under control, heat stress, and recovery conditions. **(A)** Heatmap showing expression profiles of 56 TF families across all treatments. Samples cluster primarily according to treatment rather than ploidy level. **(B)** Venn diagram illustrating the number of TF families expressed in GFD-2X and GFD-4X under heat stress and recovery conditions. **(C)** Venn diagram showing the overlap and genotype-specific expression of TF genes across treatments. **(D)** Cross-ploidy comparison of expressed TF genes under control (Mock), heat stress (HT), recovery control (R_Mock), and recovery after heat stress (R_HT) conditions. Numbers indicate shared and genotype-specific TF genes.

We next focused on TFs to characterize regulatory differences between GFD-2X and GFD-4X. Across the transcriptomic dataset, a total of 56 TF families were identified. Using a threshold of read count > 30, 1,141 TF genes were detected as expressed in GFD-2X and GFD-4X. Among these, the five most abundant TF families were bHLH, MYB, WRKY, bZIP, and ERF (**Supplementary Table 2**). Hierarchical clustering analysis demonstrated that samples grouped primarily according to treatment rather than ploidy level. Under both normal growth and high-temperature conditions, diploid and tetraploid samples clustered together within the same treatment category, indicating that environmental conditions exerted a stronger influence on global transcriptional patterns than ploidy differences. Under identical environmental conditions, GFD-2X and GFD-4X displayed broadly similar gene expression profiles (**Figure 1A**).

Venn diagram analysis further revealed that 55 TF families were expressed in both GFD-2X and GFD-4X under heat stress and recovery conditions (**Figure 1B**). Within GFD-2X, 13, 78, 16, and 15 TF genes were specifically expressed under high-temperature control, high-temperature treatment, recovery control, and recovery treatment conditions, respectively, while 744 TF genes were commonly expressed across all conditions. In contrast, GFD-4X exhibited 102, 23, 14, and 24 condition-specific TF genes under the same respective treatments, with 735 TF genes commonly expressed. These findings indicate marked differences in TF gene expression patterns between diploid and tetraploid rice during heat stress and subsequent recovery, suggesting distinct regulatory strategies in response to thermal stress (**Figure 1C**).

Direct comparisons between ploidy levels under each treatment provided further resolution. Under high-temperature control conditions, 896 TF genes were co-expressed in both genotypes, whereas 76 TF genes were uniquely expressed in GFD-4X and 50 in GFD-2X. Under heat stress treatment, 865 TF genes were shared, with 33 and 120 TF genes uniquely expressed in GFD-4X and GFD-2X, respectively. Under recovery control conditions, 823 TF genes were co-expressed, with 51 and 53 genes uniquely expressed in GFD-4X and GFD-2X, respectively. Under recovery treatment conditions, 813 TF genes were commonly expressed, while 57 were specific to GFD-4X and 30 to GFD-2X (**Figure 1D**).

When each ploidy level was analyzed independently across treatments, in the comparison groups HT_2X vs. Mock_2X and HT_4X vs. Mock_4X, 882 and 824 TF genes were co-expressed, respectively. During the recovery phase, the number of co-expressed TF genes in the comparison groups R_HT_2X vs. R_Mock_2X and R_HT_4X vs. R_Mock_4X was 777 and 800, respectively. Comparing the number of co-expressed TF genes across these two growth stages, the results indicated that the transcriptional responses of GFD-2X and GFD-4X differed in their regulatory dynamics, potentially enabling distinct transcriptional responses upon heat exposure (**Supplementary Figure 1**). During the recovery phase, shared TF expression remained substantial; however, ploidy-specific expression patterns re-emerged, implying differential transcriptional reprogramming dynamics following stress release. Collectively, these results indicate that although heat stress is the primary determinant shaping global TF expression, autotetraploid rice exhibits greater transcriptional response plasticity and expanded regulatory complexity, which may contribute to its superior thermotolerance.

### 2.2 Heat stress induces global transcriptional repression, whereas autotetraploid rice exhibits enhanced recovery-phase reactivation

**Figure 2:**
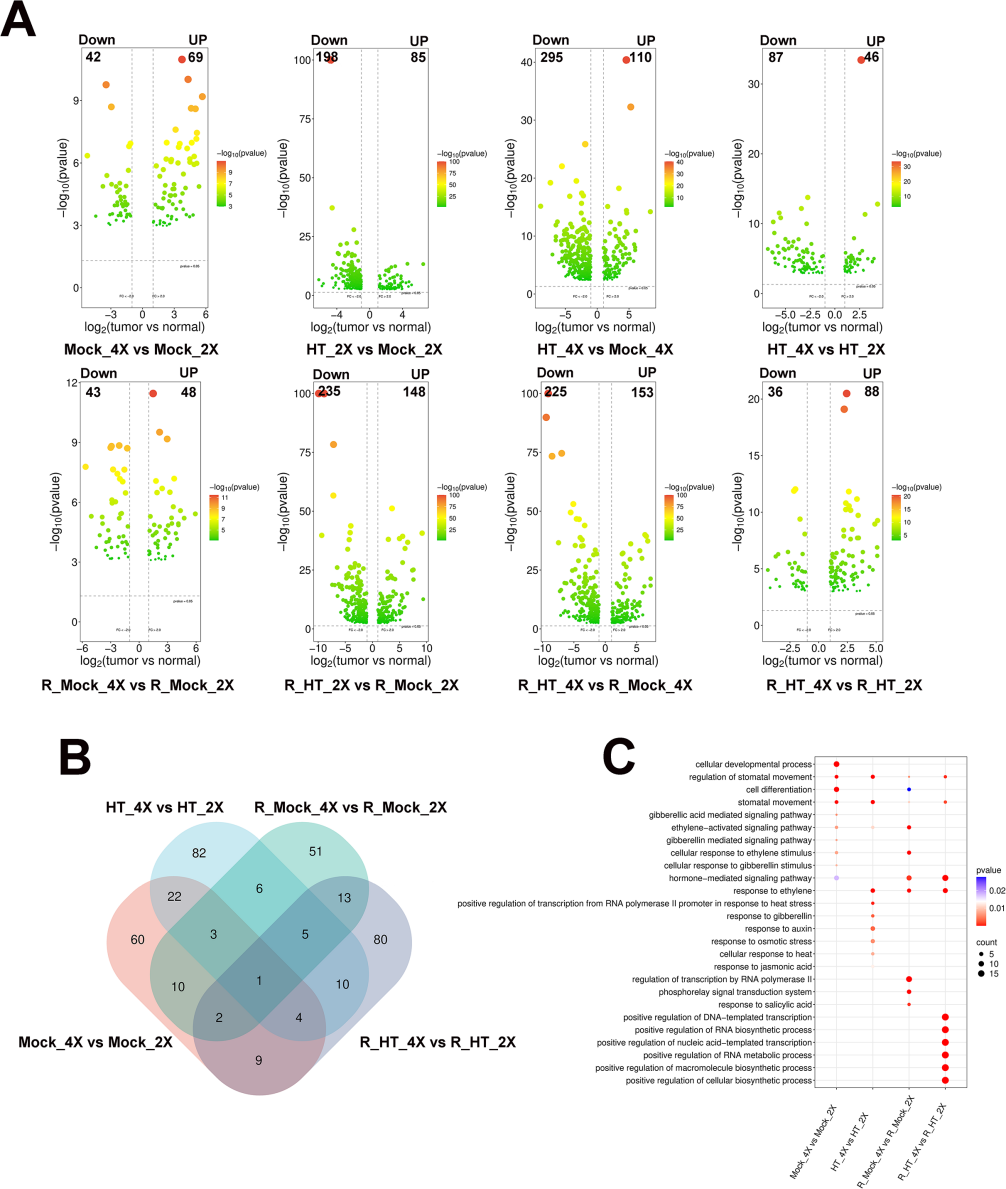
Differentially expressed transcription factor genes (DETFs) in diploid (GFD-2X) and autotetraploid (GFD-4X) rice under heat stress and recovery conditions. **(A)** Volcano plots showing DETFs between GFD-2X and GFD-4X under control, heat stress (HT), and recovery (R_HT) conditions. Red dots represent significantly upregulated genes; green dots represent significantly downregulated genes. **(B)** Venn diagrams illustrating shared and genotype-specific DETFs across different comparisons. **(C)** Gene Ontology (GO) enrichment analysis of DETFs under control, heat stress, and recovery conditions. Significantly enriched biological processes are shown (p < 0.05).

To dissect ploidy-dependent transcriptional dynamics, differentially expressed TF genes (DETFs) were identified across heat stress and recovery conditions (|log_2_FC| > 1, adjusted p < 0.05). Volcano plot analyses revealed that heat exposure triggered extensive transcriptional reprogramming in both genotypes (**Figure 2A**). During heat stress (HT vs. Mock), 198 of 283 DETFs (69.9%) in GFD-2X and 295 of 405 DETFs (72.8%) in GFD-4X were downregulated, indicating that heat stress predominantly induces transcriptional repression rather than global activation. However, quantitative differences were evident between ploidies. Under heat stress, the slightly higher upregulation ratio between GFD-2X and GFD-4X suggests there may be differences in stress response strategies between the two genotypes. During recovery (R_HT vs. R_Mock), upregulated DETFs in GFD-4X (88 genes) were more than twice the number of downregulated genes (36 genes), whereas repression remained predominant in GFD-2X (235 downregulated vs. 148 upregulated genes; 61.4% repression). In GFD-4X, upregulated DETFs substantially outnumbered downregulated DETFs, whereas repression remained relatively prominent in GFD-2X. This asymmetric reactivation pattern suggests that autotetraploid rice may possess a stronger transcriptional rebound capacity following stress removal.

Venn diagram analysis identified distinct sets of specifically expressed DETFs across the ploidy comparison groups. In Mock_4X vs. Mock_2X, HT_4X vs. HT_2X, R_Mock_4X vs. R_Mock_2X, and R_HT_4X vs. R_HT_2X, the numbers of uniquely expressed DETFs were 60, 82, 51, and 80, respectively (**Figure 2B**).

Within-ploidy comparisons assessing heat stress effects further revealed 60, 166, 48, and 62 specifically expressed DETFs in HT_2X vs. Mock_2X, HT_4X vs. Mock_4X, R_HT_2X vs. R_Mock_2X, and R_HT_4X vs. R_Mock_4X, respectively (**Supplementary Figure 2**). Notably, the tetraploid (HT_4X vs. Mock_4X) exhibited substantially more heat-responsive DETFs (166) than the diploid (60), suggesting a more extensive transcriptional reprogramming under high-temperature stress. Gene Ontology (GO) enrichment analysis highlighted functional divergence among these specifically expressed DETFs. In the Mock_4X vs. Mock_2X comparison, DETFs were primarily enriched in developmental processes, including cell differentiation (GO:0030154) and cellular developmental process (GO:0048869), reflecting intrinsic ploidy-associated developmental regulation. Under heat stress (HT_4X vs. HT_2X), DETFs were significantly enriched in three major functional categories. First, hormone signaling pathways were prominently represented, including ethylene-activated signaling pathway (GO:0009873), gibberellic acid–mediated signaling pathway (GO:0009740), and hormone-mediated signaling pathway (GO:0009755). Second, transcriptional and stress-response regulatory pathways were enriched, most notably positive regulation of transcription from RNA polymerase II promoter in response to heat stress (GO:0061408). Third, stomatal regulation–related pathways, including stomatal movement (GO:0010118) and regulation of stomatal movement (GO:0010119), were significantly enriched, indicating active modulation of gas exchange and water balance under thermal stress. During the recovery phase, functional enrichment patterns shifted. In R_Mock_4X vs. R_Mock_2X, DETFs were mainly enriched in hormone-mediated signaling pathway (GO:0009755), regulation of transcription by RNA polymerase II (GO:0006357), and phosphorelay signal transduction system (GO:0000160). In R_HT_2X vs. R_Mock_2X, DETFs were predominantly enriched in transcriptional activation–related processes, including positive regulation of DNA-templated transcription (GO:0045893), positive regulation of RNA biosynthetic process (GO:1902680), and positive regulation of nucleic acid-templated transcription (GO:1903508), while stomatal movement–related pathways (GO:0010118; GO:0010119) remained significantly enriched (**Figure 2C**).

Collectively, these data indicate that heat stress initially induces broad transcriptional modulation in both genotypes, with substantial repression and reorganization of regulatory networks. However, autotetraploid rice displays more pronounced reactivation and functional specialization of hormone-associated and transcription-related pathways during recovery, suggesting a more dynamic and resilient transcriptional reprogramming capacity that may underlie its enhanced thermotolerance.

### 2.3 Functional analysis of differentially expressed transcription factor families in GFD-2X and GFD-4X under heat stress

**Figure 3:**
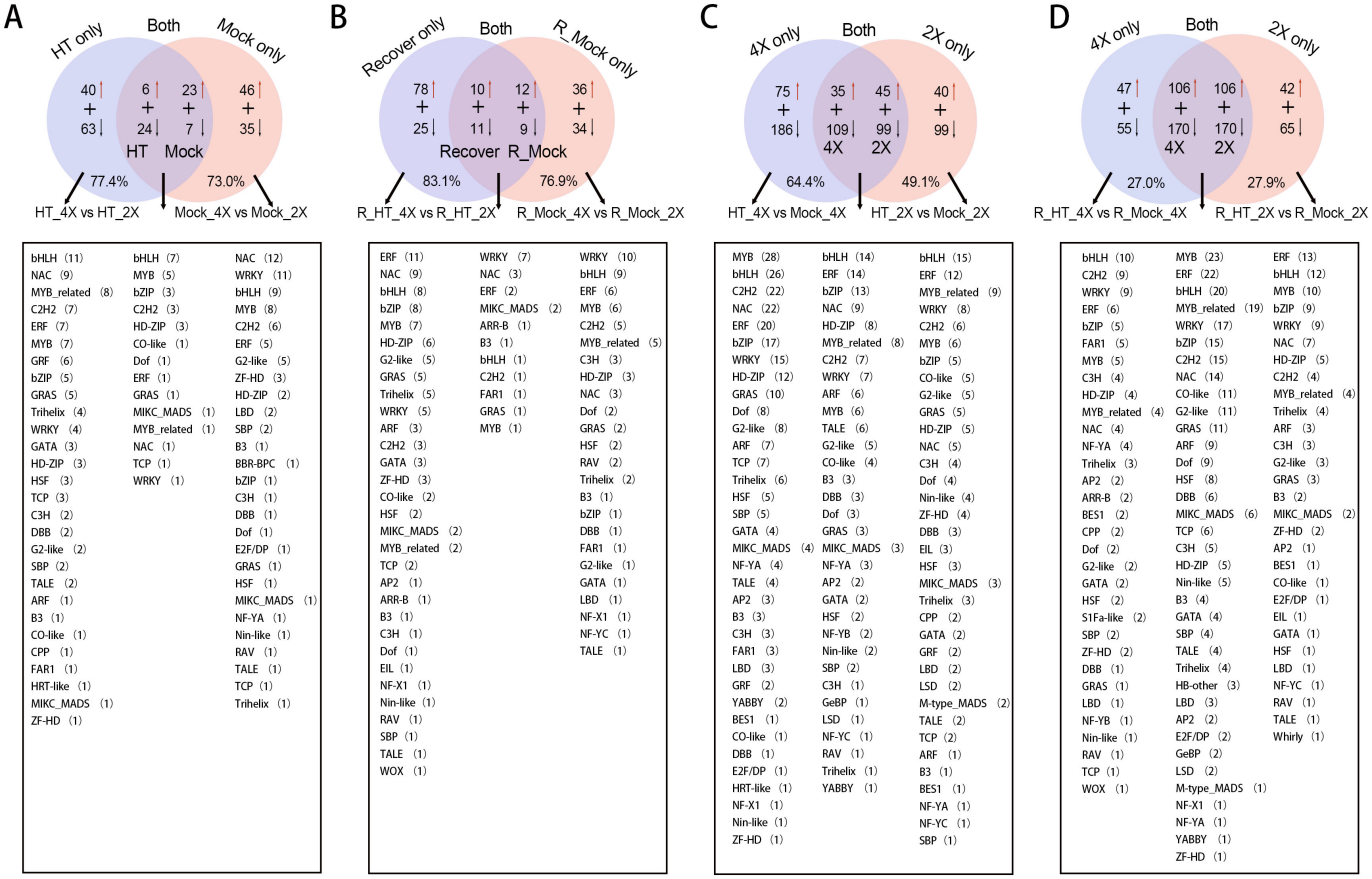
Family-level analysis of differentially expressed transcription factor genes (DETFs) under heat stress and recovery conditions. **(A)** Venn diagram summarizing stress-responsive DETFs between GFD-2X and GFD-4X under heat stress (HT) and control (Mock) conditions. **(B)** Venn diagram summarizing recovery-responsive DETFs between GFD-2X and GFD-4X under recovery (R_HT) and recovery control (R_Mock) conditions. **(C)** Ploidy-dependent DETFs identified under heat stress within each genotype (HT vs. Mock). **(D)** Ploidy-dependent DETFs identified under recovery conditions (R_HT vs. R_Mock). The distribution of major TF families (e.g., bHLH, WRKY, MYB) is indicated within each comparison.

To further dissect regulatory divergence between GFD-2X and GFD-4X rice, we compared DETFs under heat stress and recovery conditions. Under heat stress conditions, 103 DETFs were specifically detected in the HT_4X vs. HT_2X comparison, including 40 upregulated and 63 downregulated genes. In contrast, 81 DETFs were specific to Mock_4X vs. Mock_2X, comprising 46 upregulated and 35 downregulated genes. Additionally, 30 DETFs were shared between HT_4X vs. HT_2X and Mock_4X vs. Mock_2X. Among these shared genes, 6 were upregulated and 24 downregulated in HT_4X vs. HT_2X, whereas 23 were upregulated and 7 downregulated in Mock_4X vs. Mock_2X. The bHLH family represented the most abundant TF family in this comparison (**Figure 3A**). During the recovery phase, 103 DETFs were specifically expressed in R_HT_4X vs. R_HT_2X, including 78 upregulated and 25 downregulated genes. In R_Mock_4X vs. R_Mock_2X, 70 DETFs were specific (36 upregulated and 34 downregulated). A total of 21 DETFs were shared between R_HT_4X vs. R_HT_2X and R_Mock_4X vs. R_Mock_2X; in R_HT_4X vs. R_HT_2X, 10 were upregulated and 11 downregulated, whereas in R_Mock_4X vs. R_Mock_2X, 12 were upregulated and 9 downregulated. The WRKY family was the most represented TF family during recovery (**Figure 3B**). Overall, specifically expressed DETFs accounted for the majority of ploidy-differential genes under both heat stress and recovery conditions (77.4% and 73.0% under heat stress; 83.1% and 76.9% during recovery), indicating that genotype-specific transcriptional regulation predominates over shared responses between diploid and tetraploid rice.

When comparing heat treatment with control conditions within each ploidy, 261 DETFs were specifically expressed in HT_4X vs. Mock_4X (75 upregulated, 186 downregulated), whereas 139 DETFs were specific to HT_2X vs. Mock_2X (40 upregulated, 99 downregulated). A total of 144 DETFs were shared between these two comparisons; among them, 35 were upregulated and 109 downregulated in HT_4X vs. Mock_4X, while 45 were upregulated and 99 downregulated in HT_2X vs. Mock_2X. The MYB family was the most significantly represented in HT_4X vs. Mock_4X, whereas the bHLH family was prominent among shared and diploid-specific DETFs (**Figure 3C**). During recovery, 102 DETFs were specifically expressed in R_HT_4X vs. R_Mock_4X (47 upregulated, 55 downregulated), and 107 DETFs were specific to R_HT_2X vs. R_Mock_2X (42 upregulated, 65 downregulated). Notably, 276 DETFs were shared between the two recovery comparisons; in both ploidies, 106 genes were upregulated and 170 downregulated. Among these shared genes, the MYB family showed the highest representation (**Figure 3D**).

**Figure 4:**
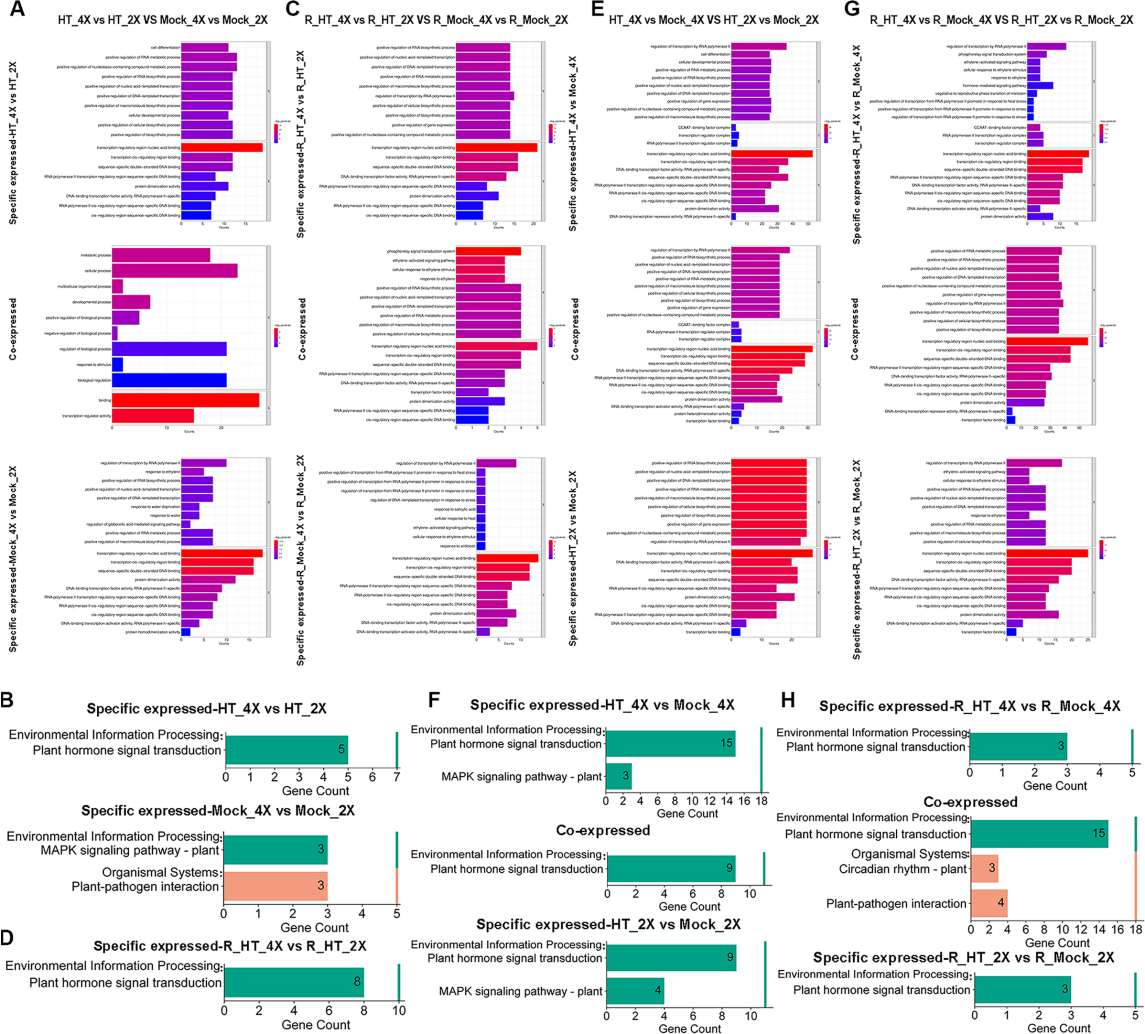
Functional enrichment analysis of differentially expressed transcription factor genes (DETFs) under heat stress and recovery conditions. **(A)** Gene Ontology (GO) enrichment analysis of DETFs between GFD-2X and GFD-4X under heat stress. **(B)** Kyoto Encyclopedia of Genes and Genomes (KEGG) pathway enrichment of heat stress-responsive DETFs. **(C)** GO enrichment analysis of DETFs under recovery conditions. **(D)** KEGG pathway enrichment of recovery-responsive DETFs. **(E, F)** GO and KEGG enrichment analyses of ploidy-dependent DETFs under heat stress. **(G, H)** GO and KEGG enrichment analyses of ploidy-dependent DETFs under recovery conditions. Significantly enriched pathways are shown (p < 0.05).

GO enrichment analysis further revealed functional divergence. In HT_4X vs. HT_2X, DETFs were significantly enriched in positive regulation of gene expression (GO:0010628) and cellular developmental process (GO:0048869). In Mock_4X vs. Mock_2X, enriched pathways included response to water deprivation (GO:0009414), response to water (GO:0009415), and regulation of gibberellic acid–mediated signaling pathway (GO:0009937). DETFs shared between these two comparisons were enriched in positive regulation of RNA metabolic process (GO:0051254), positive regulation of nucleobase-containing compound metabolic process (GO:0045935), cellular developmental process (GO:0048869), and positive regulation of macromolecule biosynthetic process (GO:0010557) (**Figure 4A**). During recovery, DETFs in R_HT_4X vs. R_HT_2X were enriched in positive regulation of nucleic acid-templated transcription (GO:1903508). In R_Mock_4X vs. R_Mock_2X, enrichment occurred in regulation of transcription by RNA polymerase II (GO:0006357), response to salicylic acid (GO:0009751), ethylene-activated signaling pathway (GO:0009873), and response to antibiotic (GO:0046677). Shared DETFs between these recovery comparisons were enriched in positive regulation of RNA biosynthetic process (GO:1902680), positive regulation of nucleic acid-templated transcription (GO:1903508), ethylene-activated signaling pathway (GO:0009873), and response to ethylene (GO:0009723) (**Figure 4C**). Within-ploidy heat responses showed that DETFs in HT_4X vs. Mock_4X were enriched in regulation of transcription by RNA polymerase II (GO:0006357), while HT_2X vs. Mock_2X showed enrichment in positive regulation of RNA biosynthetic process (GO:1902680) and transcription regulatory region nucleic acid binding (GO:0001067). Shared DETFs were enriched in regulation of transcription by RNA polymerase II (GO:0006357) and transcription regulatory region nucleic acid binding (GO:0001067) (**Figure 4E**). During recovery, R_HT_4X vs. R_Mock_4X DETFs were enriched in regulation of transcription by RNA polymerase II (GO:0006357), cellular response to ethylene stimulus (GO:0071369), response to ethylene (GO:0009723), and hormone-mediated signaling pathway (GO:0009755). R_HT_2X vs. R_Mock_2X DETFs were enriched in regulation of transcription by RNA polymerase II (GO:0006357) and ethylene-activated signaling pathway (GO:0009873). Shared DETFs were enriched in positive regulation of RNA metabolic process (GO:0051254) and regulation of transcription by RNA polymerase II (GO:0006357) (**Figure 4G**).

Kyoto Encyclopedia of Genes and Genomes (KEGG) enrichment analysis revealed that DETFs in HT_4X vs. HT_2X were significantly involved in Plant hormone signal transduction (map04075), whereas Mock_4X vs. Mock_2X DETFs were enriched in MAPK signaling pathway – plant (map04016) and Plant–pathogen interaction (map04626) (**Figure 4B**). During recovery, DETFs in R_HT_4X vs. R_HT_2X were also enriched in Plant hormone signal transduction (map04075) (**Figure 4D**). Within-ploidy comparisons showed that HT_4X vs. Mock_4X DETFs participated in Plant hormone signal transduction (map04075) and MAPK signaling pathway – plant (map04016). Shared DETFs between HT_4X vs. Mock_4X and HT_2X vs. Mock_2X were enriched in Plant hormone signal transduction (map04075), while HT_2X vs. Mock_2X–specific DETFs were enriched in MAPK signaling pathway – plant (map04016) (**Figure 4F**). Similarly, during recovery, DETFs in R_HT_4X vs. R_Mock_4X were enriched in Plant hormone signal transduction (map04075). Shared DETFs between R_HT_4X vs. R_Mock_4X and R_HT_2X vs. R_Mock_2X were enriched in Plant hormone signal transduction (map04075) and Circadian rhythm – plant (map04712). DETFs in R_HT_2X vs. R_Mock_2X were also enriched in Plant hormone signal transduction (map04075) (**Figure 4H**).

Taken together, these results demonstrate that TF reprogramming under heat stress is both genotype-specific and pathway-focused. Autotetraploid rice exhibits a greater proportion of specifically expressed DETFs, stronger enrichment in hormone signaling pathways, and broader engagement of transcriptional regulatory networks during both stress and recovery phases. In contrast, diploid rice shows relatively fewer unique TF responses and stronger reliance on MAPK-associated pathways. These findings suggest that GFD-4X may achieve enhanced thermotolerance through more complex hormone-mediated signaling integration and more extensive transcriptional modulation, enabling improved adaptive plasticity under heat stress and subsequent recovery.

### 2.4 Functional enrichment reveals a phase-dependent hormone–MAPK–transcriptional regulatory axis

To determine the biological processes underlying these transcriptional changes, GO and KEGG enrichment analyses were performed for DETFs identified under heat stress and recovery conditions (**Figure 4**). Distinct functional patterns emerged between stress and recovery phases, as well as between ploidies.

Under heat stress, DETFs were significantly enriched in hormone-mediated signaling pathways, including ethylene-activated signaling and gibberellin-mediated signaling (adjusted p < 0.05). Biological processes related to positive regulation of transcription in response to heat stress and regulation of stomatal movement were also prominently enriched. KEGG analysis further identified significant enrichment of the Plant hormone signal transduction pathway (map04075) and the MAPK signaling pathway – plant (map04016). Enrichment of MAPK signaling was particularly pronounced in genotype-dependent comparisons under heat stress, especially in GFD-4X, indicating preferential engagement of signal amplification cascades in the autotetraploid genotype. During recovery, enrichment patterns shifted toward transcriptional restoration. DETFs were significantly associated with positive regulation of RNA biosynthetic processes, positive regulation of DNA-templated transcription, and broader nucleic acid metabolic processes. KEGG enrichment consistently highlighted Plant hormone signal transduction, and shared DETFs were additionally enriched in the circadian rhythm – plant pathway (map04712), suggesting re-establishment of growth-related regulatory programs following stress removal. Direct ploidy comparisons further revealed functional asymmetry. Under heat stress, GFD-4X-specific DETFs showed stronger enrichment in MAPK signaling and hormone-related pathways than those specific to GFD-2X. During recovery, transcriptional activation-associated categories remained more prominently enriched in GFD-4X, whereas GFD-2X retained relatively stronger repression-related signatures. These results align with earlier expression analyses showing that 72.8% of DETFs were downregulated in GFD-4X during heat stress (295 of 405 genes), followed by a marked shift toward reactivation during recovery, in which upregulated DETFs (88 genes) exceeded downregulated DETFs (36 genes) by more than twofold.

Collectively, functional enrichment analyses reveal a coordinated regulatory continuum linking MAPK-mediated signal perception, hormone-dependent integration, transcriptional control, and physiological adaptation. This integrated signaling–transcriptional network appears more extensively engaged in autotetraploid rice, providing a mechanistic basis for enhanced thermotolerance and recovery capacity.

### 2.5 Co-expression network analysis identifies autotetraploid-associated regulatory modules

**Figure 5:**
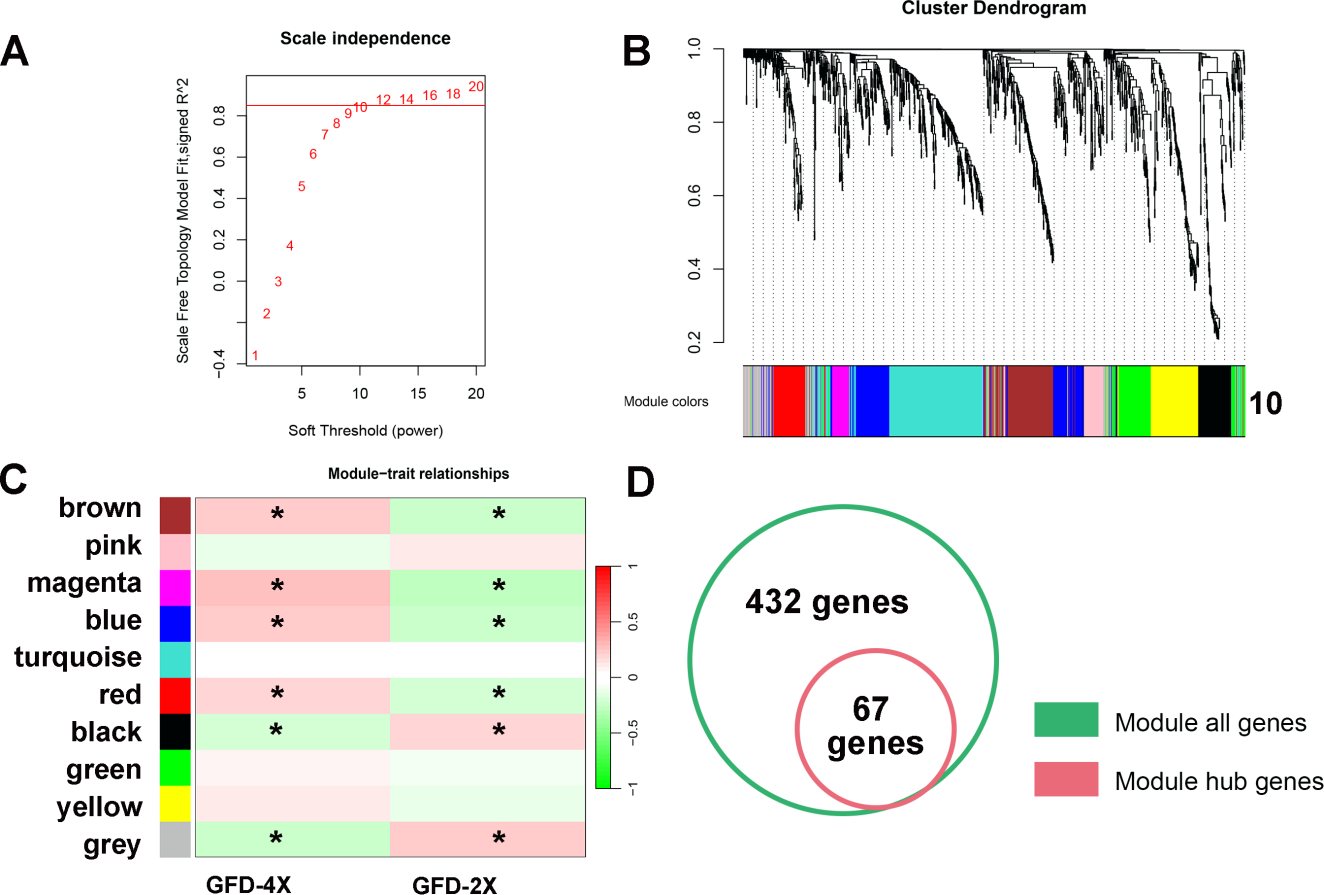
Weighted gene co-expression network analysis (WGCNA) of differentially expressed transcription factor genes (DETFs). **(A)** Scale-free topology model fit and mean connectivity analysis used to determine the optimal soft-thresholding power (β = 12). **(B)** Hierarchical clustering dendrogram of 1,141 DETFs, showing assignment into 10 co-expression modules (color-coded). **(C)** Module–trait relationship heatmap displaying correlations between module eigengenes and ploidy (GFD-2X and GFD-4X). Color intensity reflects correlation strength; values indicate Pearson correlation coefficients and associated p-values. **(D)** Identification of hub genes from the four modules (brown, magenta, blue, and red) significantly positively correlated with GFD-4X.

To assess whether these transcriptional changes are organized into coordinated regulatory networks, WGCNA (Sobhanian et al., 2026) was performed using 1,141 DETFs. A soft-thresholding power of 12 achieved scale-free topology, and hierarchical clustering partitioned DETFs into 10 co-expression modules (**Figure 5**). Module–trait correlation analysis revealed pronounced ploidy-dependent associations. The brown, magenta, blue, and red modules exhibited significant positive correlations with GFD-4X and negative correlations with GFD-2X, whereas the black and gray modules displayed the opposite trend. These four GFD-4X-associated modules collectively contained 432 genes, from which 67 hub genes were identified based on gene significance (|GS| > 0.20) and high module membership values.

The preferential association of specific modules with the autotetraploid genotype indicates that genome duplication reshapes not only individual gene expression but also higher-order regulatory architecture. Rather than acting as isolated stress-responsive elements, these hub genes are embedded within coordinated transcriptional modules that may enhance regulatory stability and integration under heat stress.

### 2.6 MAPK-associated hub transcription factors form a coordinated regulatory core

**Figure 6:**
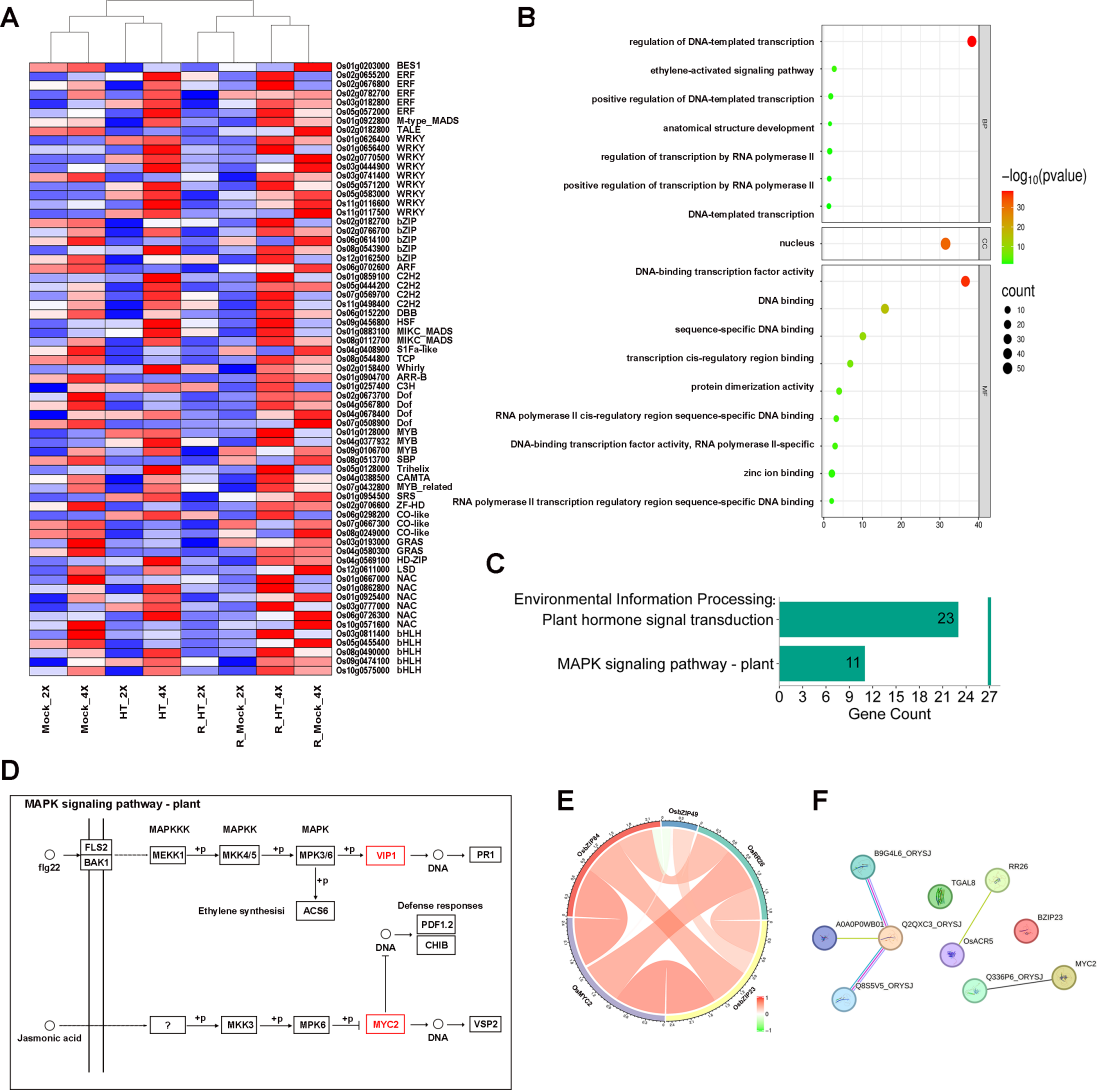
Identification and functional characterization of hub transcription factors associated with the MAPK signaling pathway. **(A)** Heatmap showing expression patterns of 67 hub genes across treatments in GFD-2X and GFD-4X. **(B)** GO enrichment analysis of hub genes. **(C)** KEGG pathway enrichment highlighting significant association with the MAPK signaling pathway – plant (map04016). **(D)** Schematic representation of hub genes mapped onto the MAPK signaling cascade. Red boxes indicate hub transcription factors. **(E)** Correlation network among five key MAPK-associated transcription factors. Edge color indicates correlation strength. **(F)** Protein–protein interaction network constructed using STRING, illustrating interactions among MAPK-associated hub genes.

To further characterize the regulatory significance of the identified hub genes, their expression patterns and functional enrichment were analyzed (**Figure 6**). Heatmap analysis demonstrated that hub gene expression levels were generally higher in GFD-4X during heat stress. During recovery, samples clustered primarily by ploidy rather than treatment, indicating divergent reprogramming trajectories between diploid and autotetraploid rice.

GO enrichment of the 67 hub genes revealed significant association with regulation of DNA-templated transcription in response to stress and RNA polymerase II-specific TF activity. KEGG pathway analysis identified significant enrichment in the MAPK signaling pathway – plant (map04016). Mapping hub genes onto this pathway demonstrated their positions downstream of MAPK-mediated phosphorylation cascades, suggesting integration of signal transduction and transcriptional regulation. Among the hub genes, five TFs—*OsbZIP23, OsbZIP49, OsbZIP84, OsRR26*, and *OsMYC2*—were identified as central components of the MAPK-associated regulatory network. *OsbZIP23, OsbZIP84*, and *OsMYC2* were significantly upregulated in GFD-4X during heat stress, whereas *OsbZIP49* and *OsRR26* exhibited preferential activation during recovery. Correlation analysis revealed strong positive associations among these five genes, and protein–protein interaction analysis further supported functional connectivity within the MAPK-centered regulatory module.

Quantitative reverse transcriptase-PCR (RT-qPCR) validation confirmed consistency between RNA-seq and RT-qPCR expression patterns (**Supplementary Figure 3**), reinforcing the reliability of the transcriptomic data. These findings identify a tightly coordinated MAPK-associated TF module preferentially activated in autotetraploid rice. This regulatory core likely functions as a central integrative hub linking stress perception, hormone signaling, and transcriptional reprogramming, thereby contributing to enhanced thermotolerance and recovery capacity in GFD-4X.

## 3 Discussion

Whole-genome duplication (WGD) is a pervasive evolutionary process that has shaped plant genomearchitecture and adaptive potential (Fan et al., 2022; Feng and Xia, 2025). Many crop species, including rice, have undergone genome duplication during their evolutionary history (Salman-Minkov et al., 2016; Ramírez-González et al., 2018) and polyploidization has been associated with enhanced tolerance to diverse abiotic stresses (Wang et al., 2022; Yong et al., 2023). While previous studies have documented physiological advantages of polyploid plants under stress conditions (López-Jurado et al., 2021; Ng et al., 2018), the transcriptional regulatory mechanisms underlying enhanced thermotolerance remain incompletely understood. In our previous study, we showed that autotetraploid and diploid rice exhibited distinct response patterns under heat stress, particularly in genes related to hormone signaling pathways, including ABA (Zhang et al., 2026). However, the expression dynamics of TFs were not examined in detail. In the present study, we further observed that, compared with hormone-related genes, TFs display more pronounced and characteristic expression patterns in response to heat stress. Our results demonstrate that autopolyploidization in rice does not merely amplify gene expression through dosage effects but instead reshapes TF-centered regulatory networks, particularly those associated with MAPK–hormone signaling and transcriptional reactivation during the recovery phase (Kazuya Ichimura et al., 2002; Rodriguez et al., 2010; He et al., 2020; Liang et al., 2024).

Heat stress is known to impair membrane stability, enzyme function, and photosynthetic processes, ultimately affecting plant growth and yield (Guilioni et al., 2003; Larkindale and Huang, 2004; Jagadish et al., 2007). In cereals, elevated temperatures during reproductive development can significantly reduce grain filling and fertility (Matsui et al., 2000; Mas‐ud et al., 2025). Plants respond to heat stress through coordinated signaling cascades that integrate stress perception with transcriptional reprogramming (Jiayu, 2025; Li et al., 2025a). Consistent with this model, our data show that heat stress triggers widespread transcriptional repression in both diploid and autotetraploid rice, with approximately 70% of DETFs downregulated during stress exposure. This global repression likely reflects energy conservation and prioritization of protective pathways. However, the magnitude and structure of transcriptional remodeling differed markedly between genotypes.

Autotetraploid rice exhibited nearly threefold more genotype-specific DETFs under heat stress compared with the diploid genotype, indicating expanded regulatory plasticity. This finding suggests that genome duplication enhances the flexibility of transcriptional responses rather than simply elevating basal expression levels. The concept of gene dosage balance predicts that duplicated regulatory elements can be selectively modulated to optimize stress adaptation (Feng and Xia, 2025). Our results support this view, demonstrating that polyploidization reshapes regulatory architecture at the network level.

A striking distinction between genotypes emerged during the recovery phase. While repression remained prominent in diploid rice, autotetraploid rice displayed pronounced transcriptional reactivation, with upregulated DETFs exceeding downregulated genes by more than twofold. Recovery capacity is increasingly recognized as a key component of stress resilience, yet it is often underexplored compared with acute stress responses. The enhanced recovery-phase reprogramming observed in GFD-4X suggests that thermotolerance involves not only improved stress perception but also accelerated restoration of transcriptional homeostasis after stress removal.

At the TF family level, distinct regulatory shifts were observed across stress phases. During heat exposure, bHLH family members were highly represented among stress-responsive DETFs. bHLH TFs have been implicated in integrating environmental cues and regulating developmental and metabolic pathways, and specific members such as CsSPT in cucumber contribute to heat tolerance by maintaining photosystem stability (Jiang et al., 2026). In contrast, WRKY TFs became dominant during recovery. WRKY proteins are widely recognized for their roles in stress signal integration and transcriptional modulation (Xie et al., 2019; Du et al., 2023), consistent with their enrichment during regulatory adjustment following stress alleviation. MYB family members were also substantially represented, aligning with previous reports that OsMYB55 enhances heat tolerance in rice by regulating proline biosynthesis and mitigating oxidative damage (Pei et al., 2017). These dynamic shifts suggest phase-specific regulatory specialization rather than uniform activation of stress-related TF families.

Functional enrichment analyses consistently identified hormone-mediated signaling and MAPK cascades as central regulatory components. Hormone pathways, including ethylene and gibberellin signaling, are key modulators of abiotic stress adaptation (Xie et al., 2019; Du et al., 2023). MAPK signaling modules are highly conserved and serve as essential conduits translating extracellular stress signals into intracellular phosphorylation events that regulate downstream TFs (Kazuya Ichimura et al., 2002; Rodriguez et al., 2010; Liang et al., 2024). In Arabidopsis, MAPK6 modulates stomatal development under heat stress through phosphorylation-dependent mechanisms (Samakovli et al., 2020; Wang et al., 2021), illustrating the integration of signal transduction and transcriptional control. The stronger enrichment of MAPK-associated processes in GFD-4X suggests enhanced signal amplification or stabilization in the autotetraploid genotype.

Network-level analysis further reinforced this interpretation. WGCNA identified four modules positively associated with GFD-4X, containing 67 hub genes embedded within coordinated regulatory networks. Such modular organization implies that heat response in autotetraploid rice is governed by integrated transcriptional programs rather than isolated gene expression events. Among the hub genes, five MAPK-associated TFs—*OsbZIP23, OsbZIP49, OsbZIP84, OsRR26*, and *OsMYC2*—emerged as central nodes. bZIP TFs are well-established regulators of abiotic stress responses (Luo et al., 2023), MYC proteins regulate stress and developmental pathways (Nongpiur et al., 2024), and OsRR26 has been implicated in stress-related signaling processes (Leng et al., 2023). The coordinated expression and interaction of these factors support the existence of a MAPK-centered transcriptional regulatory module preferentially activated in autotetraploid rice.

Collectively, these findings indicate that autopolyploidization enhances thermotolerance through multi-layered regulatory restructuring. Genome duplication expands transcriptional plasticity, reinforces MAPK–hormone signaling integration, and strengthens recovery-phase transcriptional reactivation. Rather than acting solely through gene dosage amplification, polyploidization appears to reorganize TF network topology, promoting coordinated stress adaptation.

Future studies integrating phosphoproteomic analyses and functional genetic validation will be essential to determine how MAPK-mediated phosphorylation dynamics influence these TFs and to clarify causal relationships between network restructuring and enhanced thermotolerance.

## 4 Materials and methods

### 4.1 Plant materials and growth conditions

Diploid japonica rice GFD-2X and its naturally derived autotetraploid counterpart GFD-4X were used in this study. The GFD-4X line originated from a natural mutation identified in the field from GFD-2X and was stabilized through seven generations of self-pollination, exhibiting stable agronomic traits (Xu et al., 2023; Meng et al., 2025).Uniform, full seeds of both genotypes were selected and germinated on moist filter paper for 2–3 days. Germinated seeds with consistent growth status were transferred to hydroponic culture in 0.5× Kimura B nutrient solution. Plants were grown in a controlled growth chamber under a 16 h light/8 h dark photoperiod with day/night temperatures of 26 °C/20 °C, respectively (Sanfilippo et al., 2017).

### 4.2 Heat stress and recovery treatments

At the three-leaf stage, rice seedlings with uniform growth were selected for heat stress treatment. Heat stress was applied by transferring plants to a growth chamber maintained at 40 °C for a continuous period of 7 days. Control plants were maintained under normal growth conditions (26 °C/20 °C day/night temperature with a 16 h light/8 h dark photoperiod) during the same period. Following the 7-day heat treatment, a subset of heat-treated seedlings was returned to normal growth conditions for an additional 7-day recovery period to evaluate post-stress transcriptional responses. Based on treatment conditions and sampling time points, four experimental categories were established for each genotype. The Mock group (Mock_2X and Mock_4X) consisted of plants grown under normal conditions for 7 days. The heat stress group (HT_2X and HT_4X) comprised plants exposed to 40 °C for 7 days. For recovery analysis, control plants grown continuously under normal conditions for 14 days were designated as R_Mock_2X and R_Mock_4X, while plants subjected to 7 days of heat stress followed by 7 days of recovery under normal conditions were designated as R_HT_2X and R_HT_4X.Each treatment included three independent biological replicates. For each replicate, 20 seedlings were randomly selected, and shoot tissues were harvested, immediately frozen in liquid nitrogen, and stored at −80 °C for subsequent RNA extraction and transcriptomic analysis.

### 4.3 RNA extraction, library preparation, and RNA sequencing

Total RNA was extractedusing TRIzol reagent (Invitrogen, Carlsbad, CA, USA) according to the manufacturer’s instructions. RNA concentration and purity were assessed using a NanoDrop 2000 spectrophotometer (Thermo Scientific, Waltham, MA, USA), and RNA integrity was evaluated using the Agilent 2100 Bioanalyzer system (Agilent, Waldbronn, Germany). For library construction, mRNA was purified using Dynabeads Oligo(dT) (Thermo Fisher Scientific), randomly fragmented, and reverse-transcribed into complementary DNA (cDNA). Double-stranded cDNA was synthesized, followed by end repair, A-tailing, adapter ligation, and PCR amplification according to Illumina standard protocols (Lin et al., 2022; Miao et al., 2022). Libraries were sequenced on the Illumina NovaSeq 6000 platform (Illumina, San Diego, CA, USA). Raw reads were filtered to remove adaptor sequences, reads containing poly-N, and low-quality reads to obtain clean reads (Kim et al., 2015). The overall sequencing error rate was below 0.03%, GC content exceeded 51.21%, and Q30 values were above 94.12% (**Supplementary Table 1**). Clean reads were aligned to the rice reference genome (NIP-T2T.fa; http://www.ricesuperpir.com) using HISAT2 (version 2.2.1). Gene expression levels were calculated using the FPKM (Fragments Per Kilobase of transcript per Million mapped reads) method (Sanfilippo et al., 2017; Shen et al., 2025).

### 4.4 Identification of transcription factors and differential expression analysis

Information on TF families was obtained from the Plant Transcription Factor Database (PlantTFDB; http://planttfdb.gao-lab.org/). Genes with read counts > 30 in at least one sample were retained for further analysis. Differential gene expression analysis was conducted using the DESeq2 package (version 1.22.1) in R (version 4.4.2). Genes meeting the following criteria were defined as differentially expressed genes (DEGs): |log_2_Fold Change| > 1 and adjusted p-value < 0.05. DETFs were extracted for subsequent analyses. GO enrichment analysis was performed using the DAVID database (https://davidbioinformatics.nih.gov/). KEGG pathway enrichment analysis was conducted using the Metware Cloud Platform (https://cloud.metware.cn). Statistical analyses were performed using SPSS software (version 22.0; IBM Corp., Armonk, NY, USA). Significance was determined using one-way ANOVA followed by Tukey’s test (p < 0.05).

### 4.5 Weighted gene co-expression network analysis

WGCNA was performed using the corresponding package (version 1.6.9) in R (version 4.4.2) (Langfelder and Horvath, 2008; Azam et al., 2023; Sobhanian et al., 2026). DETFs with expression variance > 0 and missing data proportion < 0.1 were included. A soft-thresholding power of 12 was selected based on scale-free topology criteria. Network construction parameters were set as follows: maximum block size = 1000, deepSplit = 4, minimum module size = 30, and mergeCutHeight = 0.25. Module eigengenes (MEs) were calculated and correlated with ploidy traits (GFD-2X and GFD-4X) using Pearson correlation analysis. Modules significantly associated with ploidy were selected for further analysis. Hub genes were defined as those with gene significance (|GS|) > 0.20 and high module membership.

### 4.6 Protein–protein interaction network construction

Protein–protein interaction (PPI) networks were constructed using the STRING database (https://cn.string-db.org/) (von Mering et al., 2003). Nodes represent known or predicted proteins, and edges represent functional associations. Network visualization was performed using online bioinformatics tools (https://www.bioinformatics.com.cn/) and R (version 4.4.2).

### 4.7 Quantitative reverse transcriptase PCR analysis

First-strand cDNA was synthesized from 1 μg of total RNA using a reverse transcription kit (Vazyme, Nanjing, Jiangsu, China) according to the manufacturer’s instructions (Caldana et al., 2007). Quantitative PCR was performed using gene-specific primers (**Supplementary Table 6**). The rice Actin gene was used as an internal reference. PCR amplification conditions were as follows: initial denaturation at 95 °C for 10 min, followed by 50 cycles of 95 °C for 25 s, 58 °C for 25 s, and 72 °C for 20 s. Relative gene expression levels were calculated using the 2^−ΔΔCt^ method. Three biological replicates were analyzed for each sample.

## 5 Conclusions

This study demonstrates that autotetraploid rice exhibits enhanced thermotolerance through coordinated remodeling of TF-centered regulatory networks. Heat stress induces widespread transcriptional repression in both diploid and autotetraploid genotypes; however, genome duplication expands stress-induced regulatory plasticity and strengthens recovery-phase transcriptional reactivation. Functional enrichment and co-expression network analyses reveal that MAPK–hormone signaling pathways form a central regulatory axis, with five MAPK-associated TFs acting as key hub genes in the autotetraploid genotype. These findings provide network-level insight into how autopolyploidization enhances stress resilience and identify candidate regulatory genes for future functional validation and breeding strategies aimed at improving heat tolerance in rice.

## Data Availability

The datasets presented in this study can be found in online repositories. The names of the repository/repositories and accession number(s) can be found below: https://www.ncbi.nlm.nih.gov/, PRJNA1424358 and PRJNA1424546.

## Supplementary material

Supplementary Table 1 Summary statistics of the RNA-seq datasets for GFD-2X and GFD-4X under heat stress treatment.

Supplementary Table 2 Differentially expressed transcription factor genes (DETFs) associated with polyploidy and heat stress after high-temperature treatment. “Y” indicates that the gene is differentially expressed; “N” indicates no differential expression.

Supplementary Table 3 Weighted gene co-expression network analysis (WGCNA) modules and the number of genes contained in each module.

Supplementary Table 4 Candidate genes screened from the brown, magenta, blue, and red modules.

Supplementary Table 5 Expression profiles of hub genes associated with the MAPK signaling pathway.

Supplementary Table 6 Primers used for quantitative reverse transcriptase PCR (RT-qPCR).

Supplementary Figure 1 Expression patterns of transcription factor (TF) genes in GFD-2X and GFD-4Xunder heat stress and recovery conditions.

Supplementary Figure 2 Functional enrichment analysis of differentially expressed TF genes (DETFs) in diploid and tetraploid rice.

Supplementary Figure 3 Validation of gene expression results by quantitative reverse transcriptase PCR (RT-qPCR).

## References

[B1] Azam M. Zhang S. Li J. Ahsan M. Agyenim-Boateng K. G. Qi J. . (2023). Identification of hub genes regulating isoflavone accumulation in soybean seeds via GWAS and WGCNA approaches. Front. Plant Sci. 14. doi: 10.3389/fpls.2023.1120498. PMID: 36866374 PMC9971994

[B2] Caldana C. Scheible W. R. Mueller-Roeber B. Ruzicic S. (2007). A quantitative RT-PCR platform for high-throughput expression profiling of 2500 rice transcription factors. Plant Methods 3, 7. doi: 10.1186/1746-4811-3-7. PMID: 17559651 PMC1914063

[B3] Du L. Huang X. Ding L. Wang Z. Tang D. Chen B. . (2023). TaERF87 and TaAKS1 synergistically regulate TaP5CS1/TaP5CR1-mediated proline biosynthesis to enhance drought tolerance in wheat. New Phytol. 237, 232–250. doi: 10.1111/nph.18549. PMID: 36264565

[B4] Fan M. Gao Y. Wu Z. Haider S. Zhang Q. (2022). Evidence for hexasomic inheritance in Chrysanthemum morifolium Ramat. based on analysis of EST-SSR markers. Genome 65, 75–81. doi: 10.1139/gen-2020-0155. PMID: 34756106

[B5] Feng K. Hou X. L. Xing G. M. Liu J. X. Duan A. Q. Xu Z. S. . (2020). Advances in AP2/ERF super-family transcription factors in plant. Crit. Rev. Biotechnol. 40, 750–776. doi: 10.1080/07388551.2020.1768509. PMID: 32522044

[B6] Feng Y. Xia P. (2025). Heat shock transcription factors as integrative hubs for plant stress adaptation: Decoding regulatory networks toward climate-resilient crop design. Plant Cell Environ. 48, 8985–9001. doi: 10.1111/pce.70185. PMID: 40947921

[B7] Guilioni L. Wéry J. Lecoeur J. (2003). High temperature and water deficit may reduce seed number in field pea purely by decreasing plant growth rate. Funct. Plant Biol. 30, 1151–1164. doi: 10.1071/fp03105. PMID: 32689097

[B8] Hamazaki N. Kyogoku H. Araki H. Miura F. Horikawa C. Hamada N. . (2021). Reconstitution of the oocyte transcriptional network with transcription factors. Nature 589, 264–269. doi: 10.1038/s41586-020-3027-9. PMID: 33328630

[B9] He X. Wang C. Wang H. Li L. Wang C. (2020). The function of MAPK cascades in response to various stresses in horticultural plants. Front. Plant Sci. 11. doi: 10.3389/fpls.2020.00952. PMID: 32849671 PMC7412866

[B10] Heslop-Harrison J. S. P. Schwarzacher T. Liu Q. (2023). Polyploidy: its consequences and enabling role in plant diversification and evolution. Ann. Bot. 131, 1–10. doi: 10.1093/aob/mcac132. PMID: 36282971 PMC9904344

[B11] Ichimura K. Shinozaki K. Tena G. Sheen J. Henry Y. Champion A. . (2002). Mitogen-activated protein kinase cascades in plants: a new nomenclature. Trends Plant Sci. 7, 301–308. doi: 10.1016/s1360-1385(02)02302-6. PMID: 12119167

[B12] Jagadish S. V. Craufurd P. Q. Wheeler T. R. (2007). High temperature stress and spikelet fertility in rice (Oryza sativa L.). J. Exp. Bot. 58, 1627–1635. doi: 10.1093/jxb/erm003. PMID: 17431025

[B13] Jiang M. García-Caparrós P. Wang Z. Zhang Y. Liao Y. Gong Y. . (2026). Evolution of mitogen-activated protein kinase in plants and AtMAPK6's role in heat stress response in Arabidopsis. Plant Sci. 362, 112767. doi: 10.1016/j.plantsci.2025.112767. PMID: 40945538

[B14] Jiayu P. (2025). The study of the alternative splicing patterns of duplicated genes on single cell level in polyploid brassica nap (Wuhan: Wuhan University of Light Industry). doi: 10.27776/d.cnki.gwhgy.2025.000005

[B15] Kim D. Langmead B. Salzberg S. L. (2015). HISAT: a fast spliced aligner with low memory requirements. Nat. Methods 12, 357–360. doi: 10.1038/nmeth.3317. PMID: 25751142 PMC4655817

[B16] Langfelder P. Horvath S. (2008). WGCNA: an R package for weighted correlation network analysis. BMC Bioinf. 9, 559. doi: 10.1186/1471-2105-9-559. PMID: 19114008 PMC2631488

[B17] Larkindale J. Huang B. (2004). Thermotolerance and antioxidant systems in Agrostis stolonifera: involvement of salicylic acid, abscisic acid, calcium, hydrogen peroxide, and ethylene. J. Plant Physiol. 161, 405–413. doi: 10.1078/0176-1617-01239. PMID: 15128028

[B18] Leng Z. Liu K. Wang C. Qi F. Zhang C. Li D. . (2023). A comparative analysis of major cell wall components and associated gene expression in autotetraploid and its donor diploid rice (Oryza sativa L.) under blast and salt stress conditions. Plants (Basel) 12, 3976. doi: 10.3390/plants12233976. PMID: 38068612 PMC10708163

[B19] Li J. Wu S. Wang K. Xu Y. Zhang X. Li X. . (2025a). A plasma membrane receptor complex mediates early heat-responsive signaling to trigger nuclear transcriptomic reprogramming in Arabidopsis. Mol. Plant 18, 2018–2034. doi: 10.1016/j.molp.2025.10.021. PMID: 41174877

[B20] Li M. Duan Z. Zhang S. Zhang J. Chen J. Song H. (2025b). The physiological and molecular mechanisms of WRKY transcription factors regulating drought tolerance: A review. Gene 938, 149176. doi: 10.1016/j.gene.2024.149176. PMID: 39694344

[B21] Liang Y. Yang C. Ming F. Yu B. Cheng Z. Wang Y. . (2024). A bHLH transcription factor, CsSPT, regulates high-temperature resistance in cucumber. Hortic. Plant J. 10, 503–514. doi: 10.1016/j.hpj.2023.02.005. PMID: 41883581

[B22] Lin Y. Ma J. Wu N. Qi F. Peng Z. Nie D. . (2002). Transcriptome study of rice roots status under high alkaline stress at seedling stage. Agronomy. 12, 925. doi: 10.3390/agronomy12040925. PMID: 41725453

[B23] López-Jurado J. Mateos-Naranjo E. Balao F. (2021). Polyploidy promotes divergent evolution across the leaf economics spectrum and plant edaphic niche in the Dianthus broteri complex. J. Ecol. 110, 605–618. doi: 10.1111/1365-2745.13823. PMID: 41875165

[B24] Luo L. Wang Y. Qiu L. Han X. Zhu Y. Liu L. . (2023). MYC2: A master switch for plant physiological processes and specialized metabolite synthesis. Int. J. Mol. Sci. 24, 3511. doi: 10.3390/ijms24043511. PMID: 36834921 PMC9963318

[B25] Mas‐ud M. A. Islam M. S. Juthee S. A. Matin M. N. Hosenuzzaman M. (2025). Mechanisms and approaches for enhancing high‐temperature stress tolerance in rice (Oryza sativa L.). J. Agron. Crop Sci. 211 (4), e70093. doi: 10.1111/jac.70093, PMID: 41940437

[B26] Matsui T. Omasa K. Horie T. (2000). High temperature at flowering inhibits swelling of pollen grains, a driving force for thecae dehiscence in rice (Oryza sativa L.). Plant Prod. Sci. 3, 430–434. doi: 10.1626/pps.3.430

[B27] Meng W. Liu Y. Zhang C. Zhan X. Wang Y. Yu X. . (2025). Integrated transcriptomic, proteomic, and metabolomic analyses reveal mechanisms underlying day-night differences in carbohydrate metabolism between diploid and tetraploid rice. Rice (N Y) 18, 65. doi: 10.1186/s12284-025-00826-z. PMID: 40668346 PMC12267728

[B28] Miao M. Tan H. Liang L. Huang H. Chang W. Zhang J. . (2022). Comparative transcriptome analysis of cold-tolerant and -sensitive asparagus bean under chilling stress and recovery. PeerJ 10, e13167. doi: 10.7717/peerj.13167. PMID: 35341039 PMC8953502

[B29] Ng D. W. Abeysinghe J. K. Kamali M. (2018). Regulating the regulators: The control of transcription factors in plant defense signaling. Int. J. Mol. Sci. 19, 3737. doi: 10.3390/ijms19123737. PMID: 30477211 PMC6321093

[B30] Nongpiur R. C. Rawat N. Singla-Pareek S. L. Pareek A. (2024). OsRR26, a type-B response regulator, modulates salinity tolerance in rice via phytohormone-mediated ROS accumulation in roots and influencing reproductive development. Planta 259, 96. doi: 10.1007/s00425-024-04366-6. PMID: 38517516

[B31] Pei G. Chen L. Zhang W. (2017). WGCNA application to proteomic and metabolomic data analysis. Methods Enzymol. 585, 135–158. doi: 10.1016/bs.mie.2016.09.016. PMID: 28109426

[B32] Ramírez-González R. H. Borrill P. Lang D. Harrington S. A. Brinton J. Venturini L. . (2018). The transcriptional landscape of polyploid wheat. Science 361, eaar6089. doi: 10.1126/science.aar6089. PMID: 30115782

[B33] Rodriguez M. C. S. Petersen M. Mundy J. (2010). Mitogen-activated protein kinase signaling in plants. Annu. Rev. Plant Biol. 61, 621–649. doi: 10.1146/annurev-arplant-042809-112252, PMID: 20441529

[B34] Salman-Minkov A. Sabath N. Mayrose I. (2016). Whole-genome duplication as a key factor in crop domestication. Nat. Plants 2, 16115. doi: 10.1038/nplants.2016.115. PMID: 27479829

[B35] Samakovli D. Tichá T. Vavrdová T. Ovečka M. Luptovčiak I. Zapletalová V. . (2020). YODA-HSP90 module regulates phosphorylation-dependent inactivation of SPEECHLESS to control stomatal development under acute heat stress in Arabidopsis. Mol. Plant 13, 612–633. doi: 10.1016/j.molp.2020.01.001. PMID: 31935463

[B36] Sanfilippo P. Miura P. Lai E. C. (2017). Genome-wide profiling of the 3' ends of polyadenylated RNAs. Methods 126, 86–94. doi: 10.1016/j.ymeth.2017.06.003. PMID: 28602807 PMC5583017

[B37] Shen Z. Jiang Z. Bai Y. Li Y. Qiao C. Jiang C. . (2025). WGCNA revealed a comprehensive gene co-expression network associated with brown spot in Nicotiana tabacum L. Ind. Crops Prod. 232. doi: 10.1016/j.indcrop.2025.121299. PMID: 41883581

[B38] Shimizu-Inatsugi R. Terada A. Hirose K. Kudoh H. Sese J. Shimizu K. K. (2017). Plant adaptive radiation mediated by polyploid plasticity in transcriptomes. Mol. Ecol. 26, 193–207. doi: 10.1111/mec.13738. PMID: 27352992

[B39] Siting W. (2025). CIPK9 and WRKY36 regulate rice disease resistancemechanism research ( Shenyang: Shenyang Agricultural University). doi: 10.27327/d.cnki.gshnu.2025.000059

[B40] Sobhanian H. Song W. Y. Soltis P. S. Soltis D. E. Chen S. (2026). Polyploidy and plant resilience to environmental stresses: Molecular mechanisms and future applications. Plant Commun., 101748. doi: 10.1016/j.xplc.2026.101748. PMID: 41620822 PMC13084105

[B41] Song Q. Chen Z. J. (2015). Epigenetic and developmental regulation in plant polyploids. Curr. Opin. Plant Biol. 24, 101–109. doi: 10.1016/j.pbi.2015.02.007. PMID: 25765928 PMC4395545

[B42] Sukumari Nath V. Kumar Mishra A. Kumar A. Matoušek J. Jakše J. (2019). Revisiting the role of transcription factors in coordinating the defense response against citrus bark cracking viroid infection in commercial hop (Humulus lupulus L.). Viruses 11, 419. doi: 10.3390/v11050419. PMID: 31060295 PMC6563305

[B43] Twyford A. D. Conover J. L. Doyle J. J. Mason A. S. Soltis D. E. Soltis P. S. . (2025). The polyploid continuum and the landscape of polyploid genomic variation. Am. J. Bot. 11, 112. doi: 10.1002/ajb2.70121. PMID: 41178014 PMC12640477

[B44] von Mering C. Huynen M. Jaeggi D. Schmidt S. Bork P. Snel B. (2003). STRING: a database of predicted functional associations between proteins. Nucleic Acids Res. 31, 258–261. doi: 10.1093/nar/gkg034. PMID: 12519996 PMC165481

[B45] Wang N. Wang S. Qi F. Wang Y. Lin Y. Zhou Y. . (2022). Autotetraploidization gives rise to differential gene expression in response to saline stress in rice. Plants (Basel) 11, 3114. doi: 10.3390/plants11223114. PMID: 36432844 PMC9698567

[B46] Wang H. Zhang Y. Norris A. Jiang C. Z. (2021). S1-bZIP transcription factors play important roles in the regulation of fruit quality and stress response. Front. Plant Sci. 12. doi: 10.3389/fpls.2021.802802. PMID: 35095974 PMC8795868

[B47] Xie Z. Nolan T. M. Jiang H. Yin Y. (2019). AP2/ERF transcription factor regulatory networks in hormone and abiotic stress responses in Arabidopsis. Front. Plant Sci. 10. doi: 10.3389/fpls.2019.00228. PMID: 30873200 PMC6403161

[B48] Xiusheng G. (2025). Study on molecular function of ZmNF-YA9 response to heat stress in maize (Yangzhou: Yangzhou University). doi: 10.27441/d.cnki.gyzdu.2025.003400

[B49] Xu M. Li D. Leng Z. Liu K. Wang C. Wang Y. . (2023). The analysis of short-term differential expression of transcription factor family genes in diploid and tetraploid rice (Oryza sativa L.) varieties during blast fungus infection. Agronomy-Basel 13, 19. doi: 10.3390/agronomy13123007. PMID: 41725453

[B50] Yong S. Lanjie Z. Chen W. Xu Z. Shengli W. (2023). Research progress on plant heat tolerance mechanisms. J. Henan Agric. Univ. 57, 713–725. doi: 10.16445/j.cnki.1000-2340.20230810.001

[B51] Zhang C. Wang Y. Meng W. Yu X. Xu M. Wang Y. . (2026). Transcriptomic and DNA methylation insights into polyploidy-enhanced heat tolerance in rice (Oryza sativa L.). Plant Physiol. doi: 10.1093/plphys/kiag135. PMID: 41845502 PMC13081708

