# Supplementary figures and images for: Autopolyploidization reshapes transcription factor regulatory networks and enhances MAPK-associated thermotolerance in rice
Source: Front Plant Sci. 2026 Apr 16;17:1813232. doi: 10.3389/fpls.2026.1813232 (PMC13128375; doi:10.3389/fpls.2026.1813232)

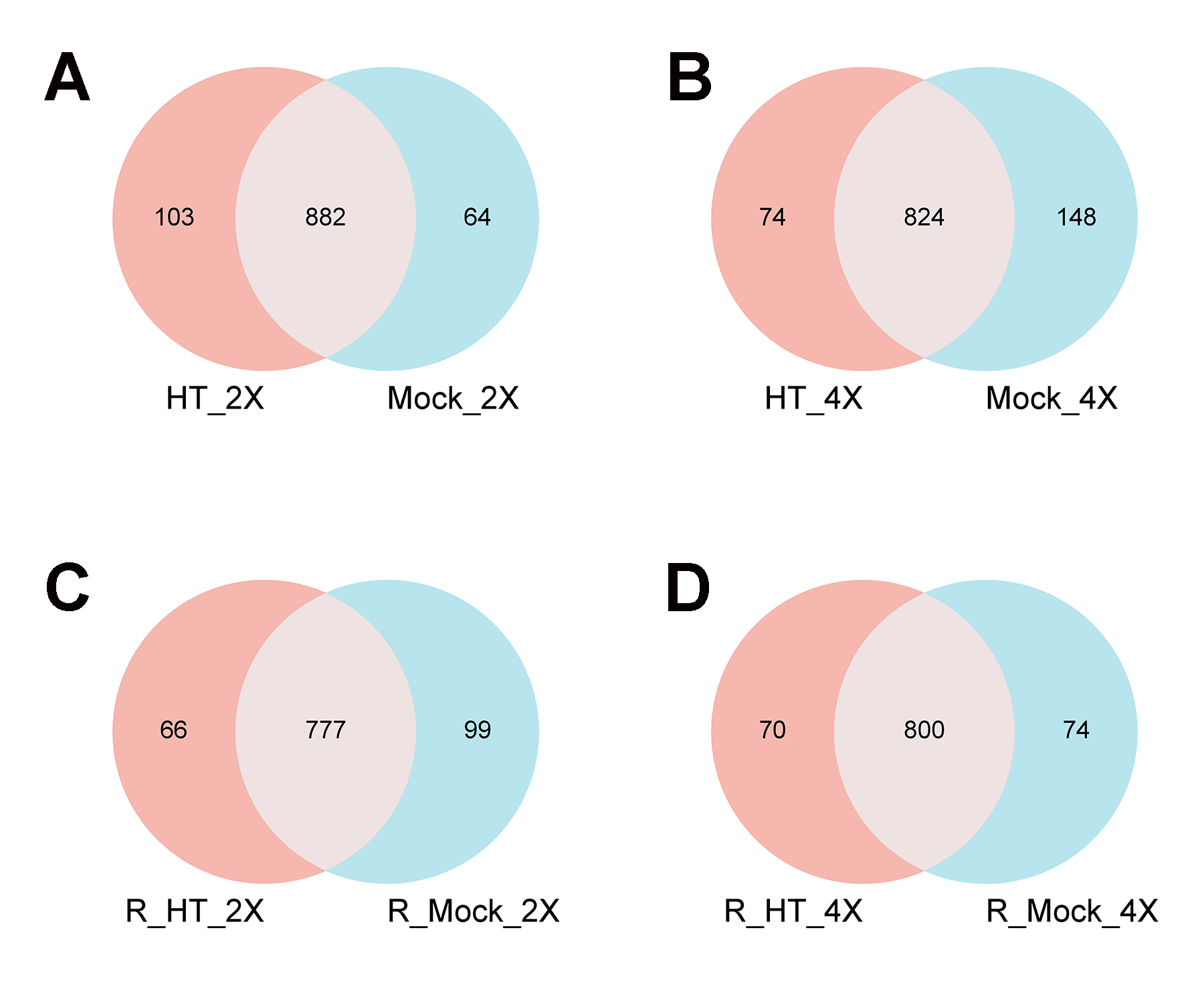

Supplement: Supplementary Figure 1 — Expression patterns of transcription factor (TF) genes in GFD-2X and GFD-4Xunder heat stress and recovery conditions. [file Image1.tif]

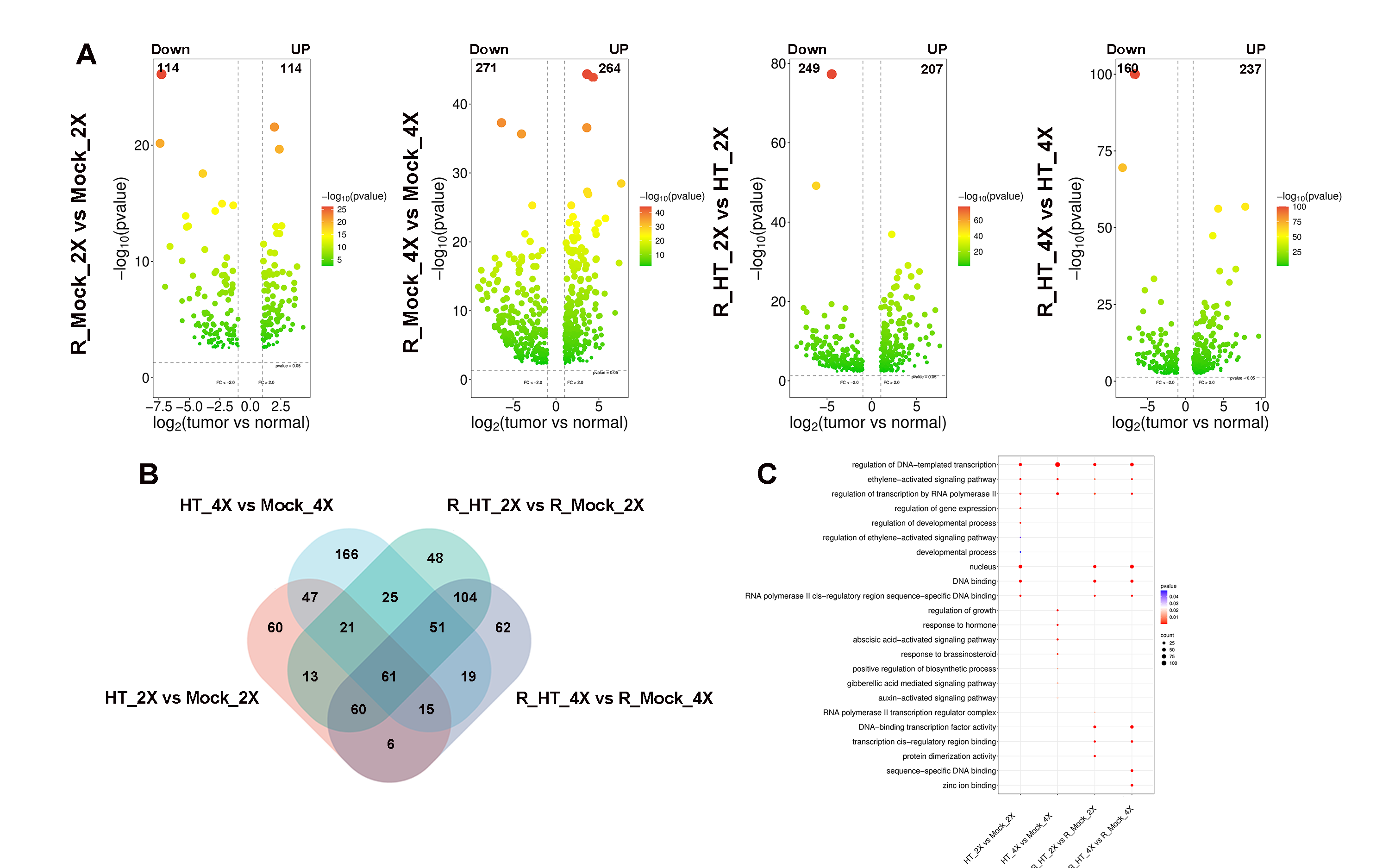

Supplement: Supplementary Figure 2 — Functional enrichment analysis of differentially expressed TF genes (DETFs) in diploid and tetraploid rice. [file Image2.tif]

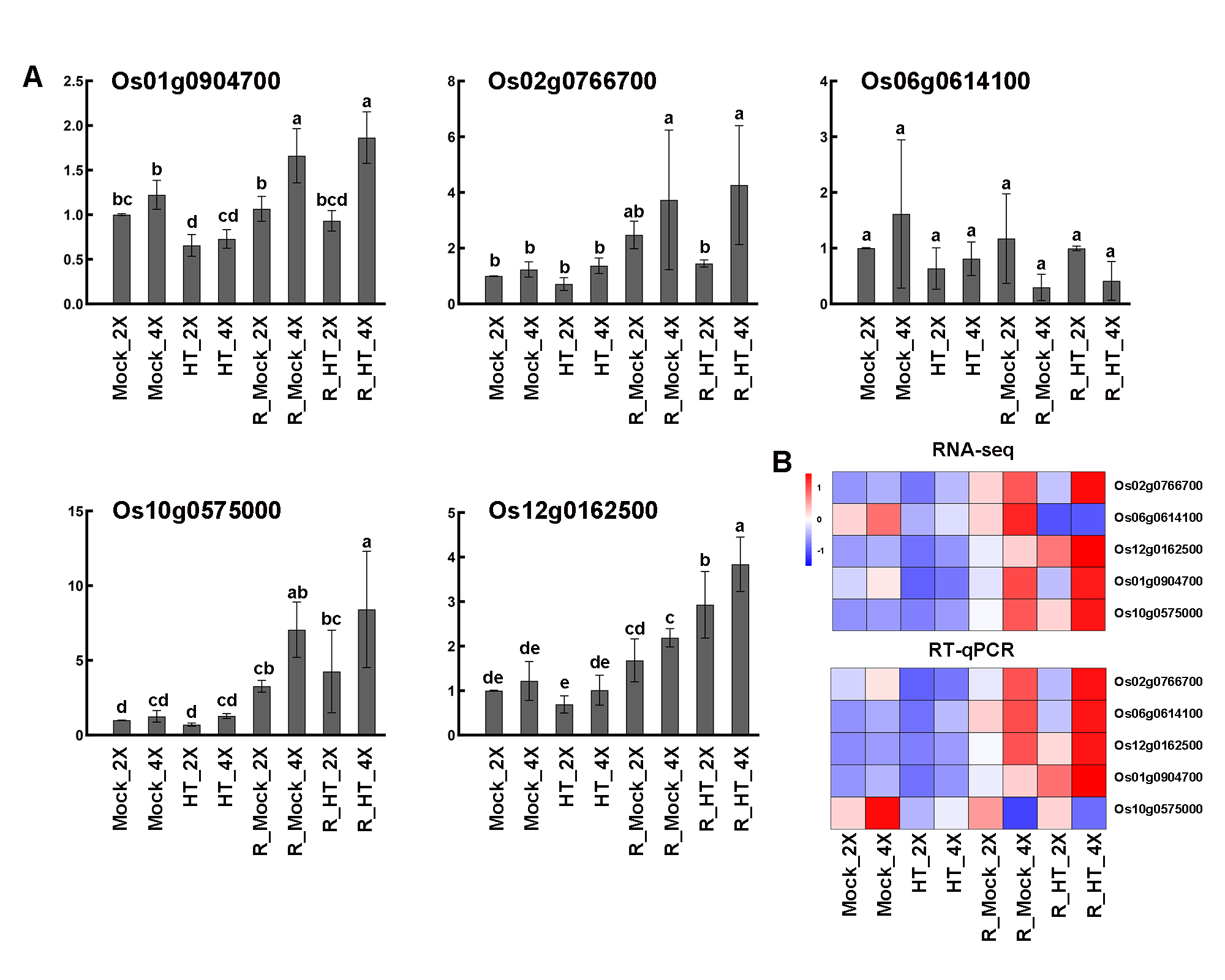

Supplement: Supplementary Figure 3 — Validation of gene expression results by quantitative reverse transcriptase PCR (RT-qPCR). [file Image3.tif]
